# Central auditory processing in teenagers with non-cholesteatomatous chronic otitis media^☆^^☆☆^

**DOI:** 10.1016/j.bjorl.2019.02.006

**Published:** 2019-04-23

**Authors:** Márcia Salgado Machado, Adriane Ribeiro Teixeira, Sady Selaimen da Costa

**Affiliations:** a Universidade Federal de Ciências da Saúde de Porto Alegre (UFCSPA), Departamento de Fonoaudiologia, Porto Alegre, RS, Brazil; b Universidade Federal do Rio Grande do Sul (UFRGS), Departamento de Fonoaudiologia, Porto Alegre, RS, Brazil; c Universidade Federal do Rio Grande do Sul (UFRGS), Departamento de Otorrinolaringologia e Oftalmologia, Porto Alegre, RS, Brazil

**Keywords:** Auditory perception, Otitis media, Auditory perception disorders, Central auditory diseases, Teenager, Percepção auditiva, Otite média, Distúrbios da percepção auditiva, Doenças auditivas centrais, Adolescente

## Abstract

**Introduction:**

Evidences of possible effects of early age otitis media with effusion in the central auditory processing, emphasize the need to consider such effects also in subjects with chronic otitis media.

**Aim:**

To investigate and analyze the impact of non-cholesteatomatous chronic otitis media on central auditory processing in teenagers.

**Methods:**

This is a study in which 68 teenagers were recruited, 34 with a diagnosis of non-cholesteatomatous chronic otitis media (study group) and 34 without otological disease history (control group). The evaluation of the subjects consisted of: anamnesis, pure-tone threshold audiometry, speech audiometry and a behavioral test battery for assessment of central auditory processing.

**Results:**

A statistically significant difference was found between the means observed in the study and control groups in all tests performed. An association was found between the control group and subgroups of the study group with unilateral alterations in all tests. An association was shown between the results for the control group and study group for family income, with a greater impact on subjects with a lower income.

**Conclusions:**

Non-cholesteatomatous chronic otitis media affects the central auditory processing in teenagers suffering from the disorder, and monaural low-redundancy hearing is the most affected auditory mechanism. Unilateral conductive changes cause more damage than bilateral ones, and lower family income seems to lead to more changes to the central auditory processing of subjects with non-cholesteatomatous chronic otitis media.

## Resumo

**Introdução:**

As evidências de prováveis efeitos de otite média com efusão precoce no processamento auditivo central, ressaltam a necessidade de se considerar tais efeitos também em sujeitos com otite média crônica.

**Objetivo:**

Investigar e analisar o impacto da otite média crônica não colesteatomatosa no processamento auditivo central em adolescentes.

**Método:**

Estudo para o qual foram recrutados 68 adolescentes, 34 com diagnóstico de otite média crônica não colesteatomatosa (grupo de estudo) e 34 sem história otológica (grupo controle). A avaliação dos indivíduos consistiu de: anamnese, audiometria do limiar auditivo para tons puros, audiometria vocal e bateria de testes comportamentais para avaliação do processamento auditivo central.

**Resultados:**

Foi encontrada uma diferença estatisticamente significante entre as médias observadas nos grupos de estudo e controle em todos os testes. Foi encontrada uma associação entre o grupo controle e os subgrupos do grupo de estudo com alterações unilaterais em todos os testes. Houve associação entre os resultados dos grupos controle e de estudo para a renda familiar, com maior impacto nos indivíduos com menor renda.

**Conclusões:**

A otite média crônica não colesteatomatosa afeta o processamento auditivo central em adolescentes, a audição monoaural de baixa redundância é o mecanismo auditivo mais afetado. Alterações condutivas unilaterais causam mais danos do que as bilaterais e a menor renda familiar parece conduzir a mais alterações no processamento auditivo central de indivíduos com otite média crônica não colesteatomatosa.

## Introduction

Central auditory processing (CAP) increases the efficiency and effectiveness with which the central nervous system uses auditory information.^1^ CAP involves a number of specific skills that are necessary for understanding what is heard.^2^ When this process is altered, difficulties in the processing of the perceived auditory information are experienced (American Speech-Language-Hearing Association, 2005), which characterize the Central Auditory Processing Disorder (CAPD).^3^

There are several known causes of CAPD, which include brain injuries, neurological disorders, and delayed maturation of the central auditory pathways.^4^ In addition to these causes, early recurrent otitis media with effusion has also been considered to be a risk factor for the development of CAPD.^2^, ^5^ It should be noted that the term “recurrent” refers to a history of six or more episodes of the disease,^6^ and the term “early” is applied when the condition occurs during the first 5 years of life.^7^, ^8^ Therefore, it is recommended that individuals with a significant history of early otitis media or other conditions that result in auditory sensory deprivation be referred for CAP assessment.^3^

Great efforts have been made to elucidate what actually occurs during the development of CAP in cases of hearing loss associated with early otitis media with effusion,^7^, ^8^, ^9^, ^10^, ^11^, ^12^, ^13^, ^14^, ^15^, ^16^, ^17^, ^18^, ^19^, ^20^, ^21^ but this has not been achieved based on the review of the literature so far.^22^

Therefore, because the evidence shows there is an association between early OME and permanent CAPD, it is reasonable to speculate that the relationship may also exist between permanent CAPD and chronic otitis media (COM), which is characterized by a chronic middle ear and mastoid infection.^23^ However, the literature only contains studies (prospective or retrospective) associating CAPD to a recurrent history of early otitis media with effusion.

Among the chronic diseases affecting the middle ear, non-cholesteatomatous chronic otitis media (NCCOM) is considered to be the most common,^24^ and it is usually accompanied with tympanic perforations or retractions as well as otorrhea and long-term hearing loss.^25^

Thus, the general objective of this study was to investigate the impact of NCCOM on central auditory processing in teenagers, to analyze the relationship between CAP and socioeconomic status, and to compare the results of CAP tests obtained in cases of unilateral and bilateral conductive defects.

## Methods

### Participants

This was a controlled, observational cross-sectional study. The sample consisted of two consecutively selected groups (non-probabilistic sample): a control group (CG) comprising adolescents without an otological disease history and a study group (SG) comprising adolescents diagnosed with NCCOM. Individuals aged between 12 and 18 years were considered adolescents.

The following inclusion criteria were used for the CG group: adolescents and attending public schools; no history of recurrent^6^ or early otitis media^7^, ^8^; normal audiological assessment (audiometry and immitanciometry); and typical overall development. The study group consisted of 34 adolescents from public schools with a diagnosis of unilateral or bilateral NCCOM who had no history of otological surgery and had mean auditory thresholds at the frequencies of 500, 1000, 2000, and 4000 Hz of up to 40 dB HL in the affected ear(s). The SG and CG groups were matched by age, sex, maternal education^26^ and family income.^27^

The following exclusion criteria were applied in both groups: presence of mental or neurological disorders or of genetic syndromes, left-handedness, history of formal music education, and presence of other risk factors for hearing loss. The information on these criteria was collected from the clinical records of each patient (study group) or through an anamnesis with their parents (control group).

The subjects in the SG were invited to participate in this study at the time of their audiological evaluation scheduled at the Speech-Language Pathology and Audiology Service, which is performed as a routine procedure at the chronic otitis media outpatient clinic of origin. The subjects of the CG were recruited from public schools. All evaluations were performed by a trained researcher experienced in conducting the study procedures. This study was evaluated and approved by the ethics committee of the institution of origin. Informed consent was obtained from all participants included in the study.

### Procedures

The following procedures were performed on the individuals in the control and study groups: anamnesis, basic audiological assessment (audiometry and speech audiometry), assessment of central auditory processing using a behavioral test battery (MLD, Masking Level Difference; SSI-ICM, Synthetic Sentence Identification with Ipsilateral Competitive Message; RGDT, Random Gap Detection Test; DPS, Duration Pattern Sequence; Musiek version and DD, Dichotic Digits Test).

The MLD was performed at the intensity of 70 dB in ears with auditory thresholds within the normal parameters (quadritonal average ≤ 25 dB) or up to 50 dB NS in ear (s) with altered thresholds (quadritonal average > 25 dB). The normality criterion used for data analysis was MLD ≥ 9 dB.^2^

The SSI–ICM was applied at an intensity of 40 dB HL in the main message, and the intensity of the ipsilateral competitive message was performed under two signal-to-noise ratio conditions (0 and −15 dB). The criteria of normality was based on the recommendations of the test authors (≥80% of correct responses for a signal-to-noise ratio of 0 dB and ≥60% of correct responses for a ratio of −15 dB).^28^

The RGDT was performed and results recorded according to the recommendations described in the test manual,^29^ and the parameter of normality used was RGDT ≤ 10 ms for subjects aged 12 or older.^2^

The DPS was performed at an intensity of 50 dB HL with a binaural presentation, and the parameter of normality used was that of at least 73% correct responses.^30^

Finally, the DD (integration stage) was performed and analyzed according to the authors’ recommendations; therefore, a percentage of correct responses of ≥95% in both ears was considered as the parameter of normality.^28^

It is noteworthy that the diagnosis of CAPD is based on the overall analysis of the results obtained in the tests selected for behavioral evaluation of CAP because these results reflect the functioning of the physiological mechanisms of the central auditory nervous system. However, for the purpose of this study, the analysis was focused on auditory mechanisms,^31^ which were evaluated by specific tests: binaural interaction (MLD), monaural low-redundancy hearing (SSI-ICM), temporal processing (temporal resolution-RGDT and temporal ordering-DPS), and dichotic hearing (DD).

### Equipment

All procedures for audiological assessment and behavioral evaluation of CAP (SG and CG) were performed in an acoustic booth using a two-channel audiometer (Interacoustics®, model AC40).

### SG subgroups

In order to analyze the impact of unilaterality and bilaterality of the conductive alterations caused by the NCCOM, the SG was divides into two groups. The first group consisted of SG subjects with Unilateral Conductive alterations (UNICON) that presented with gap without auditory thresholds sufficient for auditory hearing loss or with mild conductive hearing loss of only one ear. The second group consisted of individuals with Bilateral Conductive alterations (BILCON), that is, those who had a gap without sufficient auditory thresholds for classification of hearing loss in both ears or had mild conductive hearing loss in both ears or mixed bilateral cases characterized by one ear with a gap and the other with conductive hearing loss.

In addition to the above subgrouping, the SG sample was also stratified into five subgroups according to the type of conductive defect: Unilateral Conductive Hearing Loss (UNICHL), Bilateral Conductive Hearing Loss (BILCHL), Unilateral Gap (UNIGAP), Bilateral Gap (BILGAP) and presence of conductive hearing loss in one ear and gap in the other (MIXED).

### Sample size

The sample size was calculated using the WINPEPI software version 11.43. Using a significance level of 5%, power of 90%, and a minimum effect size of 0.8 standard deviations between the groups,^22^ the calculated minimum number of individuals per group was 33, amounting to a total of 66 individuals.

### Statistical analysis

The statistical analysis of the data was performed as described here. The quantitative variables are described as means and standard deviations or medians and interquartile ranges; the qualitative variables are described as absolute values and relative frequencies; Student's *t*-test was applied to compare means between groups; the Mann–Whitney test was used for asymmetric data; the Pearson correlation coefficient (symmetric distribution) or Spearman's rank correlation coefficient (asymmetric distribution) was applied to evaluate the association between the test results; Student's *t*-test for paired samples was used to compare the DD results between the right and left ears in the study group; and Cochran's test was used to compare changes between CAP tests in the study group. The Dunnett test was used to verify the association between the CG and the groups of unilateral, bilateral and mixed conductive changes. The significance level was set at 5% (*p* ≤ 0.05), and analyses were performed using the SPSS software version 21.0.

### Ethical considerations

This study was approved by the Research Ethics Committee of the Institution of the study, with the approval protocol n° 41689215.7.0000.5327.

## Results

The characteristics of the study participants are shown in Table 1.

**Table 1:** Characterization of the sample.

Variables	Study group (*n* = 34)	Control group (*n* = 34)	*p*
*Age (years) – average* *±* *SD*	14.9 ± 2.1	15.1 ± 2.1	0.569

*Gender – n (%)*			1.000
Male	22 (64.7)	22 (64.7)	
Female	12 (35.3)	12 (35.3)	

*Years of schooling – average* *±* *SD*	8.8 ± 1.9	9.3 ± 2.3	0.308
Age of onset OM – average ± SD	2.24 ± 1.8	–	

*Maternal schooling – n (%)*			0.452
Incomplete elementary school	15 (44.1)	11 (32.4)	
Complete elementary school	3 (8.8)	1 (2.9)	
Incomplete high school	5 (14.7)	6 (17.6)	
Complete high school	11 (32.4)	16 (47.1)	

*Family income – n (%)*			0.865
Vulnerable	16 (47.1)	17 (50.0)	
Low middle class	12 (35.3)	10 (29.4)	
Middle class middle	6 (17.6)	7 (20.6)	

*AC – average* *±* *SD*			
RE	21.2 ± 10.8	5.9 ± 3.9	<0.001^a^
LE	21.2 ± 11.4	5.7 ± 3.4	<0.001^a^

*Gap – average* *±* *SD*			
RE	19.3 ± 9.6	–	
LE	17.7 ± 9.0	–	

SD, standard deviation; *n*, number; RE, Right Ear; LE, Left Ear; AC, air conduction.

a *p* ≤ 0.05 (level of statistical significance).

All teenagers in the study group had defects in at least two physiological mechanisms of CAP. Table 2 shows a comparison of the results obtained from the CAP testing between the study group and the control group.

**Table 2:** Comparative results of CAP tests between the study and control groups.

Variables	Study group (*n* = 34)	Control group (*n* = 34)	*p*^a^	Effect size
*SSI 0 – average* *±* *SD*				
RE (%)	53.2 ± 15.5	100 ± 0.0	<0.001	4.27
LE (%)	53.5 ± 15.2	100 ± 0.0	<0.001	4.33

*SSI-15 – average* *±* *SD*				
RE (%)	29.1 ± 15.8	96.2 ± 6.0	<0.001	5.63
LE (%)	31.8 ± 15.3	95.3 ± 6.1	<0.001	5.46

*DD – average* *±* *SD*				
RE (%)	96.3 ± 4.6	99.3 ± 1.1	0.001	0.90
LE (%)	97.6 ± 3.0	99.4 ± 1.1	0.003	0.80

*DPS – average* *±* *SD (%)*	45.6 ± 22.1	61.8 ± 18.0	0.002	0.81
*MLD – average* *±* *SD (dB)*	9.2 ± 3.6	11.5 ± 1.3	0.001	0.85
*RGDT – average* *±* *SD (ms)*	14.1 ± 6.4	4.1 ± 1.6	<0.001	2.15

SD, standard deviation; *n*, number; ms, milliseconds; dB, Decibel; RE, Right Ear; LE, Left Ear; SSI, Synthetic Sentence Identification; DD, Dichotic Digits; DPS, Duration Pattern Sequence; MLD, Masking Level Difference; RGDT, Random Gap Detection Test.

a *p* ≤ 0.05 (level of statistical significance).

**Figure 1:**
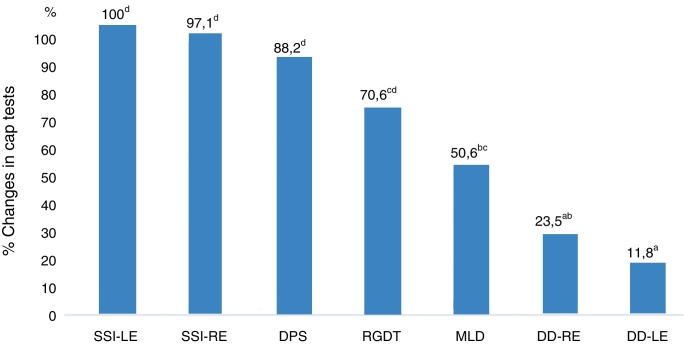
Percentage changes between CAP tests in the study group. RE, Right Ear; LE, Left Ear; SSI, Synthetic Sentence Identification; DD, Dichotic Digits; DPS, Duration Pattern Sequence; MLD, Masking Level Difference; RGDT, Random Gap Detection Test.

Fig. 1 shows the performance-related associations between the CAP tests in the study group.

With regard to the results obtained according to the type of conductive defect, the data were initially compared between the CG and SG subgroups with unilateral and bilateral conductive defects. In this analysis, a statistically significant difference was observed in all CAP tests results in a comparison of the CG with the UNICON. With regard to the comparison between the CG and the BILCON, a significant association was observed between SSI (right ear and left ear under both listening conditions), DPS, and RGDT tests results (Table 3).

**Table 3:** Comparative results of CAP tests among CG subjects and the SG subgroups with unilateral and bilateral conductive change.

Variables	Control group (*n* = 34)	UNICON subgroup (*n* = 15)	*p*^a^	BILCON subgroup (*n* = 19)	*p*
*SSI 0 – average* *±* *SD*					
RE (%)	100 ± 0.0	50.7 ± 10.3	<0.001	55.3 ± 18.7	<0.001^a^
LE (%)	100 ± 0.0	52.7 ± 10.9	<0.001	54.2 ± 18.0	<0.001^a^

*SSI-15 – average* *±* *SD*					
RE (%)	96.2 ± 6.0	27.3 ± 13.9	<0.001	30.5 ± 17.5	<0.001^a^
LE (%)	95.3 ± 6.2	28.7 ± 9.2	<0.001	34.2 ± 18.7	<0.001^a^

*DD – average* *±* *SD*					
RE (%)	99.3 ± 1.1	95.2 ± 5.9	<0.001	97.2 ± 3.1	0.054
LE (%)	99.4 ± 1.1	97.2 ± 3.4	0.004	98.0 ± 2.7	0.068

*DPS – average* *±* *SD (%)*	61.8 ± 18.0	46.4 ± 23.1	0.034	44.9 ± 21.9	0.010^a^
*MLD – average* *±* *SD (dB)*	11.5 ± 1.3	8.4 ± 3.6	0.001	9.9 ± 3.5	0.082
*RGDT – average* *±* *SD (ms)*	4.1 ± 1.6	13.0 ± 6.9	<0.001	15.1 ± 5.9	<0.001^a^

SD, Standard Deviation; *n*, number; RE, Right Ear; LE, Left Ear; ms, milliseconds; dB, Decibel; SSI, Synthetic Sentence Identification; DD, Dichotic Digits; DPS, Duration Pattern Sequence; MLD, Masking Level Difference; RGDT, Random Gap Detection Test; UNICON, Subgroup with Unilateral Conductive Change; BILCON, Subgroup with Bilateral Conductive Change.

a *p* ≤ 0.05 (level of statistical significance).

**Figure 2:**
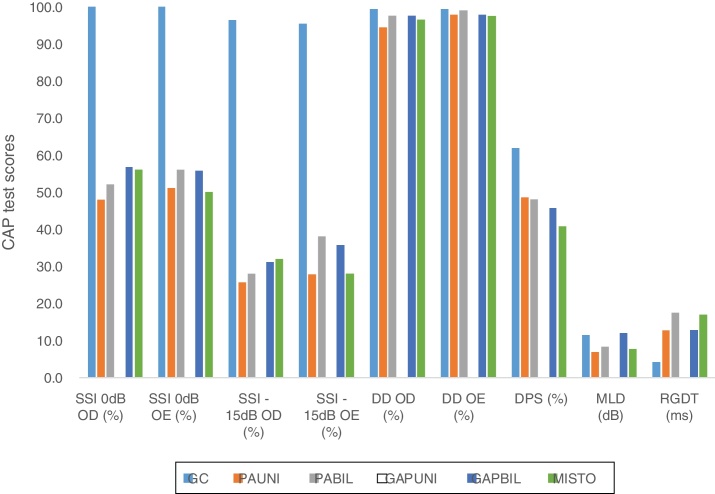
Graph showing the comparative results between the means of GC tests and SG subgroups according to the type of conductive change. CAP, central auditory processing; CG, control group; UNICHL, Unilateral Conductive Hearing Loss; BILCHL, Bilateral Conductive Hearing Loss; UNIGAP, Unilateral Gap; BILGAP, Bilateral Gap; RE, Right Ear; LE, Left Ear; SSI, Synthetic Sentence Identification; DD, Dichotic Digits; DPS, Duration Pattern Sequence; MLD, Masking Level Difference; RGDT, Random Gap Detection Test; dB, Decibels; ms, milliseconds.

In addition to the data presented, the results from the comparison between the CG and the five subgroups according to the type of the conductive defect are shown in Fig. 2.

A significant statistical difference was observed between the CG and the described subgroups with respect to the results of the following tests: SSI 0 dB and −15 dB in the right and left ears (all subgroups, *p* < 0.001), DD in the right ear (UNICHL, *p* < 0.001), DD in the left ear (UNIGAP, *p* = 0.012), MLD (UNICHL, *p* < 0.001; BILCHL, *p* = 0.037; MIXED, *p* = 0.005), and RGDT (all subgroups, *p* < 0.001). No association was found between the CG and any subgroup in the DPS.

With regard to the analysis of family income in the sample, a comparison was made between the results obtained in the CAP tests in the study and control groups for each family income range observed for the subjects in this study (vulnerable, lower middle class, and middle class) (Table 4).

**Table 4:** Comparative results of CAP tests between CG and SG according to the family income classification.

Variables	Vulnerable			Low middle class			Middle class middle		
	SG (*n* = 16)	CG (*n* = 17)	*p*^a^	SG (*n* = 12)	CG (*n* = 10)	*p*	SG (*n* = 6)	CG (*n* = 7)	*p*
*SSI 0* *dB average* *±* *SD*									
RE (%)	50.0 ± 13.2	100 ± 0.0	<0.001	60.8 ± 15.1	100 ± 0.0	<0.001^a^	46.7 ± 18.6	100 ± 0.0	<0.001^a^
LE (%)	51.9 ± 11.7	100 ± 0.0	<0.001	59.2 ± 16.2	100 ± 0.0	<0.001^a^	46.7 ± 19.7	100 ± 0.0	<0.001^a^

*SSI-15* *dB average* *±* *SD*									
RE (%)	28.8 ± 18.2	95.9 ± 6.2	<0.001	30.0 ± 11.3	94.0 ± 7.0	<0.001^a^	28.3 ± 19.4	100 ± 0.0	<0.001^a^
LE (%)	30.6 ± 12.9	95.9 ± 6.2	<0.001	35.8 ± 15.6	93.0 ± 6.7	<0.001^a^	26.7 ± 20.7	97.1 ± 4.9	<0.001^a^

*DD average* *±* *SD*									
RE (%)	94.5 ± 5.6	99.3 ± 1.2	0.005	97.5 ± 2.8	99.3 ± 1.2	0.083	98.8 ± 2.1	99.6 ± 0.9	0.368
LE (%)	97.0 ± 3.4	99.6 ± 1.0	0.011	98.1 ± 2.8	98.8 ± 1.3	0.531	98.3 ± 2.0	100 ± 0.0	0.102

*DPS (%) average* *±* *SD*	42.9 ± 19.1	62.0 ± 16.9	0.005	47.8 ± 23.3	55.3 ± 22.2	0.449	48.3 ± 29.8	70.4 ± 11.3	0.136
*MLD (dB) average* *±* *SD*	8.9 ± 4.2	11.4 ± 1.4	0.033	9.3 ± 3.2	11.6 ± 1.3	0.041^a^	10.0 ± 2.8	11.4 ± 1.5	0.270
*RGDT (ms) average* *±* *SD*	16.0 ± 7.0	3.9 ± 1.6	<0.001	12.4 ± 3.3	4.6 ± 2.1	<0.001^a^	12.8 ± 8.8	3.6 ± 0.61	0.050^a^

SG, Study Group; CG, Control Group; SD, Standard Deviation; *n*, number; ms, milliseconds; dB, Decibel; RE, Right Ear; LE, Left Ear; SSI, Synthetic Sentence Identification; DD, Dichotic Digits; DPS, Duration Pattern Sequence; MLD, Masking Level Difference; RGDT, Random Gap Detection Test; dB, Decibels.

a *p* ≤ 0.05 (level of statistical significance).

When comparing the worst mean results from the CAP tests in the CG with the best ones in the SG, a statistically significant difference was noted between the groups with respect to the SSI (*p* < 0.001) and the RGDT (*p* < 0.001) tests. However, for the other tests, this difference was not significant: DD in the right ear (*p* = 0.480), DD in the left ear (*p* = 0.551), DPS (*p* = 0.599), and MLD (*p* = 0.286).

In addition to the above analyses, the following variables were tested for associations with the CAP test results for the study group, but no association was found: sex (*p* > 0.05), age (*p* > 0.05), schooling (*p* > 0.05), age of otitis media onset (*p* > 0.05), maternal schooling (*p* > 0.10), and grade repetition (*p* > 0.2).

An analysis was also conducted to test for the presence of an advantage for the right ear in comparison with the left ear with respect to DD results, but no advantage was found (*p* = 0.08).

## Discussion

This study demonstrated that teenagers with NCCOM had significantly worse results when compared with the CG with respect to all the CAP tests performed (MLD, SSI-ICM, DPS, RGDT, and DD). However, the DD results in the study group were within the parameters of normality established for the test despite the significant difference between the groups.

With regard to the performance of the CAP tests in the study group, the SSI results were the most affected, followed by the DPS, RGDT, MLD, and DD (the least affected) results. Therefore, NCCOM caused a greater impact on monaural low-redundancy hearing and a lower impact on binaural interaction and dichotic hearing. In addition, temporal processing (temporal ordering and temporal resolution) should be noted because it also had a great impact on CAP in these subjects.

No studies assessing CAP in teenagers with NCCOM were found in the literature. Thus, the observed results were compared with the results of studies assessing CAP in children with a history of early recurrent OME.

With regard to results of the tests assessing monaural low-redundancy hearing, the literature shows similar results have been observed in subjects suffering from OME.^11^, ^17^, ^19^ In addition, a study^19^ has demonstrated that children with a history of secretory otitis media in public schools are more likely to have alterations in their auditory figure-ground ability when compared with a CG. Another study has reported that speech recognition with competition to be the task that is most sensitive to auditory deprivation.^32^

It should be noted that, in this study, the statistical difference observed in the SSI results for both ears and under the two presentation conditions (signal-to-noise ratio 0 dB and −15 dB) showed a very strong effect size, i.e., >1.30.^33^ Thus, there is no doubt about the difficulty that these subjects face in monaural low-redundancy listening (auditory figure-ground and auditory closure skills). These skills are crucial in the classroom,^2^, ^11^, ^34^ which is an environment that does not provide favorable conditions for hearing due to noise, reverberation, and the distance between the student and the teacher.^34^ Therefore, it is difficult for students to ignore sounds that interfere with the teacher's speech (fans, steps in the hallway, classmates talking, honks, or noises from the street).^2^, ^11^, ^34^ This hampers the understanding of what is being said and possibly impairing the learning.^2^, ^19^, ^34^

With regard to binaural interaction, other studies have already shown alterations in children with a history of OME,^9^, ^12^, ^13^, ^14^, ^15^, ^21^ which are findings that support the results found in this study. On the other hand, some studies have reported that OME does not compromise binaural interaction^10^; the binaural interaction is compromised only when there was a high incidence of the disease during their first five years of the subject's life^7^; or binaural interaction is recovered in adolescence.^12^ In this study, the strong correlation between MLD results and binaural interaction (ES = 0.85) allows us to infer that, in NCCOM, binaural interaction is altered.

With regard to temporal processing, several studies have already shown impairment in children with a history of early OME.^16^, ^19^, ^20^, ^22^ A study^19^ found that temporal resolution and ordering were the most affected abilities in subjects with a history of secretory otitis media from public schools. A greater probability of having these abilities impaired was found in subjects with the disease when they were compared with subjects in the CG. On the other hand, one study^8^ has suggested that temporal resolution becomes normal following the recovery of tonal thresholds, demonstrating that this aspect still raises doubts when early OME is concerned. In this study, temporal processing proved to be one of the most affected mechanisms, and the effect size observed in data analysis was considered to be “very strong” (ES = 2.15) in the RGDT (temporal resolution) and “strong” (ES = 0.81) in the DPS (temporal ordering).

It should be noted that, with respect to DPS testing, the values observed in the CG were below the parameters of normality stipulated by the test author. Therefore, this was a more difficult test when compared to the temporal ordering test Pitch Pattern Sequence (PPS), which has been mostly used to assess this skill.^35^ However, because of the careful selection of the subjects who made up the CG in this study, these values are believed to be possibly associated with individual socioeconomic status; this is because temporal ordering tests may be influenced by the intellectual ability of the subjects.^36^ Thus, further studies are needed to analyze the parameters of normality of the DPS across different age groups and socioeconomic levels in the Brazilian population.

With respect to binaural integration, a number of studies have shown a right ear advantage in dichotic listening in children with a history of early OME,^14^, ^15^, ^18^ a fact that is explained by the maturational delay probably arising from the inconsistency of auditory stimulation caused by fluctuating hearing loss associated with early otitis media with effusion.^19^ In this study, such an advantage was not observed, which was expected due to the age of the subjects in the sample (≥12 years old) because the maturation of auditory perception remains stable from age 10/12 years.^37^ In addition, the results of this study are consistent with the significantly lower DD results observed when compared to a CG.^19^, ^22^

Although the average percentage of correct answers in the DD testing in the study group showed normality in both ears in this study, a significant difference, with a strong effect size, was found in the SG compared to the CG. This observation cannot be ignored; therefore, the mechanism of binaural integration is believed to be probably impaired in adolescents with NCCOM.

With regard to the characteristics of conductive hearing loss caused by NCCOM, some aspects need to be scrutinized. The association observed between the results of all CAP tests in the CG and in the groups with unilateral defect and bilateral conductive defects demonstrates that both (unilateral defect and bilateral defects) change auditory perception at the level of the CANS. However, unilateral cases are clearly more compromised.

Therefore, when the brain is deprived of binaural input and receives only monaural stimulation, the cortex undergoes reorganization in the ensuing years.^38^ In addition, unilateral hearing loss known to affect not only auditory functions – such as localizing sounds and listening to noise^17^ – but also the development of speech and language,^39^ behavior, and school performance.^40^ These changes seem to occur due to a restriction to signal input across the bilateral auditory pathways, possibly leading to permanent reorganization. Thus, the unilateral input strengthens the auditory pathways on the stimulated side, while the developmental pathways of the deprived ear remain immature or are subject to degenerative changes or reorganization.^38^

Furthermore, regarding the effects of unilateral conductive hearing loss, an animal model study has also demonstrated alterations in binaural hearing,^41^ which was explained by the attenuation and delay in sound conduction, which would cause distortions of the acoustic cues used for sound localization and other aspects of binaural hearing. Consequently, it seems that the central auditory system responds dynamically to the level of neural input received from the ears.^42^

Thus, there is evidence that the conductive hearing loss present in otitis media can cause asymmetry to the auditory levels of the ears, as in cases of unilateral conductive change, which would result in a negative effect on the complex auditory processing,^43^ as demonstrated in this study.

In the analysis of the subgroups stratified by type of conductive alteration (unilateral or bilateral), it was observed that the SSI and RGDT tests (monaural low-redundancy hearing and temporal processing) were affected in the same way regardless of the extent of the resulting conductive defect (unilateral vs. bilateral; mild conductive hearing loss vs. presence of gap without degree of loss). These results support the data obtained from the analysis of performance of the CAP tests in the SG.

Additionally, the analysis showed there was significant association between DD results and exclusively unilateral alterations, indicating the relevance of binaural hearing for the ability of binaural integration as well. On the other hand, MLD results were significantly associated with alterations in all subgroups with hearing loss (UNICHL, BILCHL, and MIXED), which reinforces the influence that this test usually has detecting peripheral conductive and/or sensorineural hearing losses.^44^

Therefore, this study points out a very relevant aspect. It should be noted that the changes observed in this study were not limited to cases of conductive hearing loss, but also to cases of unilateral and bilateral gap. Emphasis should be given to unilateral cases, which, at the clinic, are generally disregarded for referral for assessment and therapeutic rehabilitation on the grounds that the contralateral ear is normal. However, as shown in this study, these cases seem to have a greater impact on central auditory processing than bilateral cases; therefore, they must not be ignored.

It is evident that further research is needed to elucidate the mechanism related to unilateral and bilateral conductive changes and chronic otitis media and central auditory processing. However, this first piece of evidence is worthy of consideration and further investigation.

The relevance of the socioeconomic status in otitis media is well known. However, it has not been discussed extensively in the literature, probably because it is a field that is not within the expertise of biomedical researchers.^45^ The data on maternal schooling and family income were considered in this study to be socioeconomic indicators, the analysis of their correlations with CAP in the adolescents in the sample was performed. Maternal schooling showed no association with CAP results. On the other hand, some results related to family income showed significant correlations; for example, all SG subjects with a “vulnerable” family income had significantly worse results in all CAP tests when compared to individuals with the same income. These associations were also observed in three tests (SSI, MLD, and RGDT) in subjects who were classified in the “lower middle class” stratum in both the control and study groups and only in two tests (SSI and RGDT) in the “middle middle class” subgroup. Thus, the data suggest that changes seem to be less evident as family income increases.

It should be noted that the SSI and RGDT tests showed significant differences between the CG and SG across all family income strata, and these data are consistent with results of the analysis of the percentage of defects seen in each test because they were the first and third most affected tests. It is also noteworthy that the DD and DPS tests, which require greater cognitive demand due to the CANS structures involved in the binaural integration and temporal ordering mechanisms, showed an association between the groups only for the “vulnerable” subgroup. Accordingly, the socioeconomic status, in this study represented by family income, seems to interfere negatively with the central auditory processing of adolescents with NCCOM.

We also investigated whether the worse results obtained in the CG could be similar to the better results in the SG. However, this similarity was observed only in the DD, DPS and MLD tests, but it was not found in the SSI and RGDT tests, which is in agreement with the other analyses.

Therefore, based on these results, most of the central nervous system was found to be functionally compromised in adolescents with NCCOM; this is because the CAP tests used in this study to assess each of the auditory mechanisms are sensitive to the following injuries: lower half of the brainstem (binaural interaction),^35^ brainstem and primary auditory cortex (monaural low-redundancy hearing),^46^ intra-hemispheric and inter-hemispheric (temporal ordering) connections,^2^ and primary auditory cortex (temporal resolution).^47^

Thus, the initial hypothesis that chronic otitis media would affect CAP was confirmed. This effect of CAP is related to the severity and duration of the symptoms that occur with the disease. In this context, it is essential to mention the continuum theory^48^ that depicts otitis media as a series of continuous events that can start as secretory otitis media and evolve (if untreated or if there is no spontaneous regression) into chronic otitis media.^49^ Therefore, adolescents diagnosed with NCCOM have probably had changes in the middle ear, even if subtle, over the course of their development.

These alterations are believed to have interfered with the proper development of the central auditory nervous system of the adolescents in this study due to deleterious effects on the quality of auditory signals^35^, ^50^ that have occurred in the middle ear since childhood. In addition, the presence of fluid in the middle ear can also interfere with speech perception,^37^ even in the absence of a relevant auditory alteration.

In view of the results found, the relevance of this study should be highlighted because it is an unpublished work that was carefully designed and was based on a reference service for treatment of chronic otitis media. The representative sample and the high power of the statistical data used in data analysis, which are reflected in the intensity of CAPD in the tested teenagers, are also noteworthy.

However, it is crucial to note that further clarification is necessary on the mechanism of dichotic hearing in this population because the adolescents in the study group had normal scores, which casts doubts over the impact of NCCOM on integration. For the other mechanisms assessed, their impacts on CAP were evident.

This study demonstrated that NCCOM can affect CAP in adolescents. Therefore, clinicians caring for this group must be urged to refer these individuals for CAP assessment and for adequate therapeutic intervention, with a view to a better quality of life for them. Further studies are suggested on individuals with chronic otitis media (cholesteatomatous and non-cholesteatomatous) in order to broaden the knowledge about its impact on CAP and to allow the professionals concerned to properly refer this population as necessary.

## Conclusions

The results of this study demonstrate that NCCOM causes changes in the following physiological mechanisms of central auditory processing: binaural interaction, temporal processing, and monaural low-redundancy hearing, the latest being the most affected. Furthermore, central auditory processing is affected by unilateral and bilateral conductive defects associated with NCCOM, and the effect is greater in unilateral cases. Family income seems to be a socioeconomic indicator associated with the worsening of CAPD in patients with NCCOM.

## Conflicts of interest

The authors declare no conflicts of interest.

## References

[1] American Speech-Language-Hearing Association. Central auditory processing disorders [Technical Report]; 2005. Available from: www.asha.org/policy [accessed 27.02.17].

[2] Ramos B.D., Costa-Ferreira M.I.D., Guedes M.C., Alvarez A.M., Junior D.C., Burns D.A.R., Lopez F.A. (2014). Tratado de pediatria: sociedade brasileira de pediatria.

[3] American Academy of Audiology. Clinical Practice Guidelines: Diagnosis, treatment and management of children and adults with central auditory processing disorder; 2010. Available from: www.audiology.org [accessed 27.02.17].

[4] Pereira L.D., Bevilacqua M.C. (2013). Tratado de audiologia.

[5] Carvalho N.G., Novelli C.V.L., Colella-Santos M.F. (2015). Fatores na infância e adolescência que podem influenciar o processamento auditivo: revisão sistemática. Rev CEFAC.

[6] Howie V.M., Ploussard J.H., Sloyer J. (1975). The ‘otitis-prone’ condition. Am J Dis Child.

[7] Hogan S.C.M., Moore D.R. (2003). Impaired binaural hearing in children produced by a threshold level of middle ear disease. J Assoc Res Otolaryngol.

[8] Hartley D.E.H., Moore D.R. (2005). Effects of otitis media with effusion on auditory temporal resolution. Int J Pediatr Otorhinolaryngol.

[9] Moore D.R., Hutchings M.E., Meyer S.E. (1991). Binaural masking level differences in children with a history of otitis media. Int J Audiol.

[10] Hutchings M.E., Meyer S.E., Moore D.R. (1992). Binaural masking level differences in infants with and without otitis media with effusion. Hear Res.

[11] Gravel J.S., Wallace I.F. (1992). Listening and language at 4 years of age: effects of early otitis media. J Speech Hear Res.

[12] Hogan S.C.M., Meyer S.E., Moore D.R. (1996). Binaural unmasking returns to normal in teenagers who had otitis media in infancy. Audiol Neurotol.

[13] Hall J.W., Grose J.H., Dev M.B., Drake A.F., Pillsbury H.C. (1998). The effect of otitis media with effusion on complex masking tasks in children. Arch Otolaryngol Head Neck Surg.

[14] Asbjornsen A., Holmefjord A., Reisaeter S., Moller P., Klausen O., Pritz B. (2000). Lasting auditory attention impairment after persistent middle ear infections: a dichotic listening study. Dev Med Child Neurol.

[15] Klausen O., Moller P., Holmefjord A., Reisaeter S., Asbjornsen A. (2000). Lasting effects of otitis media with effusion on language skills and listening performance. Acta Otolaryngol.

[16] Sandeep M., Jayaram M. (2008). Effect of early otitis media on speech identification. Aust New Zeal J Audiol.

[17] Keogh T., Kei J., Driscoll C.J., Khan A. (2009). Children with minimal conductive hearing impairment: speech comprehension in noise. Audiol Neurotol.

[18] Lima-Gregio A.M., Calais L.L., Feniman M.R. (2010). Otitis media and sound localization ability in preschool children. Rev CEFAC.

[19] Borges L.R., Paschoal J.R., Colella-Santos M.F. (2013). Central auditory processing: the impact of otitis media. Clinics.

[20] Villa P.C., Zanchetta S. (2014). Auditory temporal abilities in children with history of recurrent otitis media in the first years of life and persistent in preschool and school ages. CoDAS.

[21] Tomlin D., Rance G. (2014). Long-term hearing deficits after childhood middle ear disease. Ear Hear.

[22] Khavarghazalani B., Farahani F., Emadi M., Dastgerdi Z.H. (2016). Auditory processing abilities in children with chronic otitis media with effusion. Acta Otolaryngol.

[23] Bluestone C.D., Paparella M.M., Shumrick D.A. (1991). Otolaryngology. Volume II: otology and neuro-otology.

[24] Caldas N., Caldas N., Neto S.C., Sih T. (1999). Otologia e audiologia em pediatria.

[25] Silva M.N.L., Selaimen F., Piltcher O.B., Costa S.S., Maahs G.S., Kuhl G.(org) (2015). Rotinasemotorrinolaringologia.

[26] Brasil. Lei 9394, de 20 de Dezembro de 1996. Lei de Diretrizes e Bases da EducaçãoNacional. Brasília: Presidência da República; 1996.

[27] Secretaria De Assuntos Estratégicos, Presidência Da República. Relatório de definição da classe média no Brasil; 2012. Available from: http://www.sae.gov.br/documentos/publicacoes/relatorio-de-definicao-da-classe-media-no-brasil [accessed 23.01.15].

[28] Pereira L.D., Schochat E. (2011).

[29] Keith R.W. (2000).

[30] Musiek F. (1994). Frequency (pitch) and duration pattern tests. J Am Acad Audiol.

[31] Academia Brasileira de Audiologia. Fórum de diagnóstico audiológico. 31° Encontro Internacional de Audiologia. São Paulo, SP; 2016. Available from: http://www.audiologiabrasil.org.br/31eia/pdf/forum_f.pdf [accessed 28.02.17].

[32] Schilder A.G.M., Snick A.F.M., Straatman H., Broek V.D. (1994). The effect of otitis media with effusion at preschool age on some aspects of auditory perception at school age. Ear Hear.

[33] Rosenthal J.A. (1996). Qualitative descriptors of strength of association and effect size. J Soc Sci Res.

[34] Katz J., Ferre J., Keith W., Alexander A.L., Katz J., Chasin M., English K., Hood L.J., Tillery K.L. (2015). Handbook of clinical audiology.

[35] Bellis T.J. (2003).

[36] Delecrode C.R., Cardoso A.C.V., Frizzo A.C.F., Guida H.L. (2014). Pitch pattern sequence and duration pattern tests in Brazil: literature review. Rev CEFAC.

[37] Katz J., Tillery K.L., Lichtig I., Carvallo R.M.M. (1997). Audição: abordagens atuais.

[38] Gordon K.A., Wong D.D.E., Papsin B.C. (2013). Bilateral input protects the cortex from unilaterally-driven reorganization in children who are deaf. Brain.

[39] Vieira M.R., Nishihata R., Chiari B.M., Pereira L.D. (2011). Perception of limitations on communicative activities, temporal resolution and figure-to-ground in unilateral hearing loss. Rev Soc Bras Fonoaudiol.

[40] Tibbetts K., Ead B., Umansky A., Coalson R., Schlaggar B.L., Firszt J. (2011). Interregional brain interactions in children with unilateral hearing loss. Otolaryngol Head Neck Surg.

[41] Polley D.B., Thompson J.H., Guo W. (2013). Brief hearing loss disrupts binaural integration during two early critical periods of auditory cortex development. Nat Commun.

[42] Moore D.R., Hartley D.E.H., Hogan S.C.M. (2003). Effects of otitis media with effusion (OME) on central auditory function. Int J Pediatr Otorhinolaringol.

[43] Williams C.J., Jacobs A.M. (2009). The impact of otitis media on cognitive and educational outcomes. Med J Aust.

[44] Jerger J., Brown D., Smith S. (1984). Effect of peripheral hearing loss on the MLD. Arch Otolaryngol.

[45] Bluestone C.D., Klein J.O., Paradise J.L., Eichenwald H., Bess F.H., Downs M.P. (1983). Workshop on effects of otitis media on the child. Pediatrics.

[46] Sanchez M.L., Alvarez A.M.M.A., Costa S.S., Cruz O.L.M., Oliveira J.A.A.(org) (2006). Otorrinolaringologia: princípios e prática.

[47] Costa-Ferreira M.I.D., Cardoso M.C.(org) (2015). Fonoaudiologia na infância: avaliação e terapia.

[48] Paparella M.M., Hiraide F., Juhn S.K., Kaneco J. (1970). Cellular events involved in middle ear fluid production. Ann Rhinol Otol Laryngol.

[49] Costa S.S., Dornelles C.C., Netto L.F.S., Braga M.E.L., Costa S.S., Cruz O.L.M., Oliveira J.A.A.(org) (2006). Otorrinolaringologia: princípios e prática.

[50] Talaat H.S., Kabel A.H., Qatanani F.E. (2009). Paediatric speech intelligibility (PSI) in normal hearing children with history of recurrent otitis media with effusion (OME). Audiol Med.

